# Double-edged sword: flavivirus NS1 enhances human plasmin-mediated fibrinolysis while being targeted for cleavage and inactivation

**DOI:** 10.1099/jgv.0.002253

**Published:** 2026-04-29

**Authors:** Lucas Mendes-Monteiro, Pedro Henrique Carneiro, Jonas N. Conde, Joice L. Menezes, Matheus Silva de Souza, Pedro F. de Carvalho, Eugenio D. Hottz, Eva Harris, Diego Allonso, Ronaldo Mohana-Borges

**Affiliations:** 1 Instituto de Biofísica Carlos Chagas Filho, Universidade Federal do Rio de Janeiro, Rio de Janeiro, Brazil; 2 Division of Infectious Diseases and Vaccinology, School of Public Health, University of California, Berkeley, CA, USA; 3 Laboratório de Imunotrombose, Departamento de Bioquímica, Universidade Federal de Juiz de Fora, Juiz de Fora, Brazil; 4 Departamento de Biotecnologia Farmacêutica, Faculdade de Farmácia, Universidade Federal do Rio de Janeiro, Rio de Janeiro, Brazil; 5 Sanford Consortium for Regenerative Medicine, University of California, San Diego, CA, USA

**Keywords:** dengue, fibrinolysis, flavivirus, non-structural protein 1 (NS1), plasminogen, plasmin

## Abstract

Dengue virus (DENV) is the most prevalent arbovirus in the world, being transmitted to humans through the bite of infected *Aedes* mosquitoes. Symptomatic individuals may develop a haemorrhagic fever leading to plasma leakage, hypovolaemic shock and death. A key player in viral pathogenesis is the non-structural protein 1 (NS1), which is secreted by infected cells and disrupts platelet and endothelial barrier function, contributing to plasma extravasation. Importantly, dengue patients present abnormal haemostasis, with concomitant activation of both coagulation and fibrinolytic systems correlating with disease severity. However, a direct role played by NS1 in fibrinolysis has never been shown. Here, we investigated the interaction between NS1 and plasminogen, the precursor of the fibrinolytic enzyme plasmin, aiming to characterize its implications in clot turnover during infection. We showed that binding of plasminogen to NS1 is dependent on lysine-binding sites in plasminogen, implicating NS1 as a plasmin substrate. We also demonstrated that NS1 is cleaved by plasmin, which, in turn, blocks its effect on endothelial glycocalyx layer disruption and endothelial hyperpermeability. Using euglobulin clot lysis assays, we showed that DENV NS1 enhances fibrin clot lysis and that this effect is conserved for other flaviviruses, including Zika and West Nile viruses. Altogether, we identified a novel mechanism by which NS1 might contribute to bleeding disorders, highlighting the relevance of the plasminogen/plasmin system for DENV pathogenesis in humans.

## Introduction

Dengue is an arthropod-borne disease caused by dengue virus (DENV), which belongs to the *Flaviviridae* family and coexists as four distinct serotypes [1]. It is estimated that 390 million infections occur annually, rendering DENV a major challenge to global public health [2]. Patients usually develop flu-like symptoms, in some cases progressing to severe manifestations characterized by haemorrhage and shock [3].

The DENV genome encodes a polyprotein that is processed into structural and non-structural proteins, among which the non-structural protein 1 (NS1) is the only one secreted to the extracellular milieu [4,5]. Outside the cell, NS1 contributes to pathogenesis by numerous mechanisms. For instance, NS1 promotes endothelial cell glycocalyx (EGL) disruption through heparanase activation and induced expression of sialidases [6], as well as mislocalization of tight and adherens junction proteins [7] and the induced release of vasoactive proinflammatory cytokines from leukocytes [8], leading to plasma extravasation *in vitro* and *in vivo* [8,9]. Moreover, NS1 expression in DENV-infected platelets leads to activation through an autocrine loop, paving the way for thrombocytopenia and haemorrhage [10]. Remarkably, NS1 was also shown to inhibit prothrombin activation, evidencing an anticoagulant activity [11].

Aiming to unravel DENV NS1’s multiple roles during infection, our group has previously identified 52 liver proteins that bind NS1 by yeast two-hybrid screening [12]. Among them, human plasminogen stood out as an interesting partner, since it is the zymogen form of the serine protease plasmin. Plasmin acts mainly on fibrinolysis but also contributes to angiogenesis, tissue remodelling and inflammation [13,15]. The direct involvement of plasmin in DENV infection and transmission was evidenced by increased viral replication in the gastrointestinal tract of infected *Aedes aegypti* mosquitoes fed with high-plasmin-containing blood [16]. Plasmin is recruited to the site of infection by the Envelope (E) protein to trigger mucosal glycocalyx degradation, thereby facilitating DENV internalization [16]. Moreover, direct plasminogen activation by E protein was correlated with disease severity in humans, highlighting the relevance of plasminogen/plasmin regulation in pathogenesis [17].

NS1 is an indispensable player in disease progression, modulating both endothelial function and haemostasis and contributing to severe dengue [18]. Additionally, although fibrinolysis dysregulation was evidenced in dengue patients and was correlated with bleeding complications [19,20], a direct role played by NS1 in the fibrinolytic system and its main effector, plasminogen/plasmin, is yet to be described. Here, we identify human plasminogen as a novel interaction partner for NS1, implicating a new role for NS1 in dengue disease. Altogether, our results demonstrate that despite not modulating plasminogen activation directly, NS1 is targeted as a substrate by plasmin, leading to viral protein cleavage and attenuation of key effects attributed to NS1 during infection, namely EGL disruption and endothelial hyperpermeability. Furthermore, we show that DENV NS1 enhances plasmin-mediated fibrinolysis, which is also conserved for other flaviviruses, including Zika and West Nile viruses (ZIKV and WNV, respectively). Therefore, we describe a previously unrecognized conserved profibrinolytic effect resulting from NS1 release by flavivirus-infected cells.

## Methods

### Proteins

Recombinant DENV2 or ZIKV NS1 protein expressed in *Escherichia coli* (non-glycosylated dimer) was produced as previously described [21]. Alternatively, HEK293-derived glycosylated DENV2 (Thailand strain 16681), ZIKV (Uganda strain) and WNV NS1 were acquired from The Native Antigen, being certified to be endotoxin-free and >95% purity.

### Enzyme-linked immunosorbent assay (ELISA)

Human plasminogen (Sigma #SRP6518) or BSA were adsorbed (10 µg ml^−1^) on the wells of microplates (Nunc) overnight at 4 °C and blocked in 2% BSA, 0.05% Tween 20 PBS-T (pH 7.4) solution for 1 h at 37 °C. Serial dilutions (25–0 µg ml^−1^) of non-glycosylated dimeric DENV2 NS1 were added to wells and incubated for 2 h at 37 °C. For competitive ELISA, plasminogen was adsorbed (100 nM) and incubated with fixed non-glycosylated DENV2 NS1 concentrations (25 µg ml^−1^), prior to addition of serial *ε*-aminocaproic acid (EACA; Sigma #A2504) dilutions (100–0 mM) across the plate. Plates were washed thrice with PBS-T, followed by anti-DENV2 mouse polyclonal antibody [21] labelling for 1 h at 37 °C. After repeating the washing step, HRP-conjugated anti-mouse IgG antibody (Invitrogen #62-6520; 0.02 µg ml^−1^) was incubated for 1 h at 37 °C. A 5 mg tablet of o-phenylenediamine dihydrochloride (Sigma-Aldrich) was dissolved in 0.05 M phosphate-citrate buffer (pH 5.0), followed by the addition of H_2_O_2_. This solution was applied to each well, and plates stood by at room temperature for ~7 min, being protected from light. Stop solution (H_2_SO_4_ 9 N) was added, and the result was obtained through a spectrophotometer (SpectraMax M5; Molecular Devices) at a wavelength of 492 nm. To determine whether glycosylation played a role in the interaction between NS1 and plasminogen, bacteria or HEK293-derived ZIKV NS1 were coated (10 µg ml^−1^) on microplate wells and incubated with increasing concentrations of human plasminogen (25–0 µg ml^−1^). Binding was detected with anti-human plasminogen (Abcam #ab154560; 0.07 µg ml^−1^) and developed as described above.

### Molecular docking

Modelling of the interaction was performed using the HADDOCK server (high ambiguity protein-protein docking) version 2.4 [22,23], based on the crystallized structure of the glycoform I of human plasminogen (PDB accession number 4DUU) [24] and the dimeric DENV2 NS1 (16681 strain; PDB accession number 4O6B) [25]. Standard default settings were used; residues of the lysine-binding site in kringle 1 (Asp137, Asp139 and Arg153) were chosen to guide docking, whereas lysine residues on DENV2 NS1 were selected as candidate interaction determinants. Structural visualization and analysis were performed using the UCSF Chimera software version 1.19 (University of California San Francisco) [26].

### Plasminogen activation assay

Human plasminogen (100 nM) and urokinase plasminogen activator (uPA, Sigma #SRP6273; 20 nM) were incubated in the presence of either glycosylated DENV2 NS1 or BSA (10 µg ml^−1^ each) in the wells of microplates (Kasvi). Alternatively, EACA (1 mM) was incubated as a positive control. Afterwards, the chromogenic plasmin substrate S-2251 (Chromogenix #S820332; 200 nM) was added to a final volume of 100 µl, and the absorbance kinetics at 405 nm was detected over 1 h by the SpectraMax M5 (Molecular Devices). As a negative control, a condition containing plasminogen and S-2251 but lacking uPA was prepared, as well as another condition in which only PBS was added to plasminogen, uPA and S-2251.

### Flavivirus NS1 cleavage assays

Human plasminogen (1 µM) was incubated with uPA (200 nM) and glycosylated DENV2 NS1 (1.83 µM) for 4 h at 37 °C. Aliquots were obtained every hour for subsequent western blot analysis with anti-NS1 antibody [21]. As negative controls, samples containing plasminogen and uPA, or only NS1, or NS1 and uPA/plasminogen were used, and all were incubated under the same experimental conditions.

For the evaluation of differential cleavage between DENV2 and ZIKV NS1, viral proteins (1.83 µM) were incubated with human plasmin (Abcam #90928; 1 µM) for 45 min at 37 °C. Samples were collected every 15 min for subsequent analysis by western blot, using anti-ZIKV NS1 polyclonal antibody (Genetex #133307; 0.5 µg ml^−1^).

### Western blot

Polyacrylamide gels and nitrocellulose membranes were pre-wetted in a 30 g l^−1^ Tris, 144 g l^−1^ glycine, 20% methanol solution, followed by transfer in the Trans-Blot Semi-Dry device (BioRad) for 1 h under a constant voltage of 15 V. Blots were blocked in 5% skim milk in TBS-T (0.05% Tween 20 in TBS; 25 mM Tris/HCl, 3 mM KCl, 140 nM NaCl, pH 7.4) for 1 h at room temperature. Primary antibodies were incubated overnight at 4 °C under gentle rocking. Unbound immunoglobulins were washed out with TBS-T (three rounds of 10 min incubations under agitation) prior to the addition of HRP-conjugated anti-mouse or rabbit IgG secondary antibody (0.25 or 0.375 µg ml^−1^, respectively). Chemiluminescence was generated with the Pierce Fast Western Blot Kit, SuperSignal West Pico Substrate (Thermo Fisher Scientific) and detected by the ImageQuant LAS 4000 or 500 device (GE Healthcare).

### *In silico* prediction of plasmin cleavage sites

The primary sequence of DENV2 NS1 protein (UniprotKB accession number DENV-2 P29991) was analysed using the getMerops SitePrediction server [27] with default settings. Candidate cleavage sites were scored based on experimentally defined plasmin specificity, as determined by substrate phage display, positional-scanning synthetic combinatorial libraries and solution-phase fluorogenic peptide microarray approaches [28,30]. A similar method has been previously used for plasmin target motif prediction [31]. For top hits selection, some criteria were established: (1) the residue at position P1 should be either lysine or arginine, (2) the average score should be higher than 1 and (3) predicted fragments should match western blot observations. For the investigation of differential plasmin cleavage, DENV2 and ZIKV (UniprotKB Q32ZE1) NS1 sequences were aligned and analysed using the PRALINE software [32].

### Cell culture

Human pulmonary microvascular endothelial cells (HPMEC, clone ST1-6R) were maintained using endothelial cell basal medium-2 supplemented with growth factors: FBS (5%), hydrocortisone (0.2 ml), R3-IGF-1 (0.5 ml), ascorbic acid (0.5 ml), hEGF (2 ml) and gentamicin-1000 (0.5 ml) as per the manufacturer’s specifications (Lonza). HPMECs were a gift from Dr J.C. Kirkpatrick, Johannes Gutenberg University, Germany.

### Fluorescence microscopy

For the analysis of EGL degradation, HPMECs were grown on sterile coverslips coated with 0.2% gelatin (Sigma) and imaged on a Zeiss LSM 710 Axio Observer inverted fluorescence microscope (CRL Molecular Imaging Center, UC Berkeley). Recombinant DENV2 NS1 protein (10 µg ml^−1^) was preincubated with human plasmin (1 µM) for 2 h at 37 °C and added to confluent cell monolayers. For sialic acid staining, wheat germ agglutinin (WGA) conjugated to Alexa Fluor 647 (5 μg ml^−1^; Sigma) was incubated 6 h post-treatment (hpt) with live cells (300 µl) for 30 min at 37 °C and 5% CO_2_. Untreated monolayers were used as a control for normal expression/distribution of sialic acid. Next, coverslips were rinsed twice with PBS and fixed with 4% formaldehyde for 20–30 min at 4 °C, protected from light. Hoechst 33342 (0.125 μl per slide; Invitrogen) was used for nuclei staining, whereas ProLong^®^ Gold Antifade (Invitrogen) was used as mounting medium. Images were acquired at 20× magnification and were processed and analysed with ImageJ software. All RGB images were converted to grayscale. Then, mean grayscale values and integrated density were taken and plotted as mean fluorescence intensity (MFI). MFI was measured using ImageJ [33].

### Transendothelial electrical resistance

The effect of DENV2 NS1 on endothelial permeability was evaluated by measuring the transendothelial electrical resistance (TEER) of endothelial cell monolayers grown on a 24-well Transwell polycarbonate membrane system (Transwell^®^ permeable support, 0.4 µm, 6.5 mm insert; Corning, Inc.). Briefly, HPMECs were seeded on the apical side of Transwell inserts (top chamber) in a final volume of 300 µl per well. Each Transwell was transferred to a 24-well plate containing 1.5 ml of endothelial cell culture medium, representing the basolateral side of each Transwell. Transwells containing HPMECs were incubated at 37 °C and 5% CO_2_ for 3 days, and 50% of culture medium was changed in each well 48 h post-seeding. Cells were grown until TEER values between 150 and 180 Ω were reached, indicating 100% cell confluency. Recombinant DENV2 NS1 (5 µg ml^−1^) was preincubated with human plasmin (1 µM) for 30 min and added to the apical side of the Transwell insert containing the cell monolayer. TEER values, expressed in Ω, were collected at the indicated time points following the addition of test proteins using an Epithelial Volt Ohm Meter with ‘chopstick’ electrodes (World Precision Instruments). Endothelial permeability was expressed as relative TEER, which represents a ratio of resistance values (Ω) as follows: (Ω experimental condition−Ω medium alone)/(Ω non-treated endothelial cells−Ω medium alone).

### Euglobulin clot lysis assay

To assess the role of flavivirus NS1 on fibrinolysis, the euglobulin clot lysis assay was adapted from a previous publication [34]. Peripheral venous blood was obtained from healthy donors, as approved by the Institutional Review Board of the Federal University of Juiz de Fora (HU-UFJF, 2.223.542). Informed volunteers consented to experimental procedures and data publication. Briefly, samples were collected into tubes containing 0.129 M sodium citrate (pH 6.1; 9:1 blood-sodium citrate ratio) and centrifuged at 2,500 ***g*** for 20 min at 4 °C. Supernatant was recentrifuged under the same conditions for poor-platelet plasma (PPP) harvesting. For euglobulin fraction formation, PPP was precipitated with 0.017% acetic acid (0.35 ml:6.3 ml) in a 13×100 mm glass tube, being incubated on ice for 10 min and centrifuged at 2,000 ***g*** for 5 min at room temperature. Supernatant was removed and the remaining liquid was drained out. Pellet was resuspended in a 154 mM NaCl, 2.6 mM sodium borate solution (pH 9.0; 350 µl), being stirred with a glass Pasteur pipette and placed once in a water bath at 37 °C for complete dissolution. Samples were applied to prewarmed 96-well plates in duplicates in the presence or absence of glycosylated DENV2, ZIKV or WNV NS1 (5 µg ml^−1^) at a final volume of 150 µl. As a negative control, the same volume of Dulbecco’s PBS (DPBS; CaCl_2_ 0.9 mM, KCl 2.68 mM, MgCl_2_•6 H20 0.49 mM, NaCl 136.8 mM, Na_2_HPO_4_ 15.2 mM) was added. A 0.025 M CaCl_2_ (150 µl) solution was added to a single well of each sample to induce coagulation. Remaining wells were left as blank controls without Ca^2+^ addition. Turbidimetry kinetics was assessed by the SpectraMax M5 (Molecular Devices) at 405 nm for 720 min, measuring absorbance every 3 min. Data were analysed as described [34].

### Statistical analysis

Statistical significance was achieved when *P* values were <0.05. Multiple comparisons were performed using one-way ANOVA with Tukey’s or Dunnett’s post-test, and asterisks in the figures indicate significant differences relative to the control, as indicated in the legends (*, *P*<0.05; **, *P*<0.01; ***, *P*<0.001; ****, *P*<0.0001). Analyses were performed using Prism version 10 (GraphPad Software).

## Results

### DENV2 NS1 binds human plasminogen in a lysine-dependent manner

**Fig. 1.**
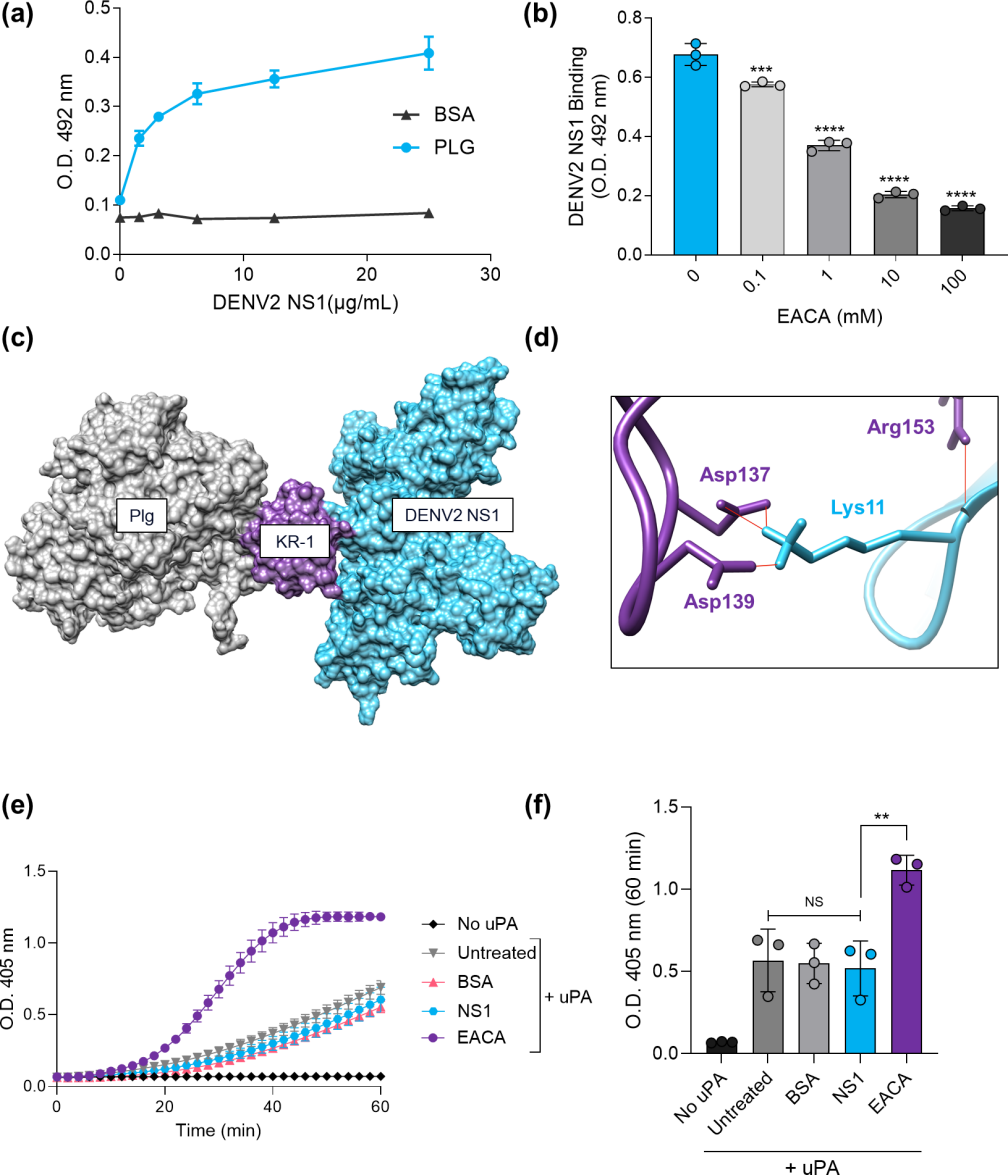
DENV2 NS1 binds human plasminogen in a lysine-dependent manner but does not alter plasminogen activation by uPA. (**a**) Human plasminogen (Plg) or BSA (10 µg ml^−1^) were coated on microplate wells and incubated with increasing concentrations of non-glycosylated dimeric DENV2 NS1 (0–25 µg ml^−1^). Binding was detected by anti-DENV2 NS1 antibody labelling, followed by absorbance detection at 492 nm. Data are presented as mean±sd from two independent experiments. (**b**) Plasminogen was coated (100 nM) and incubated with fixed concentrations of non-glycosylated dimeric DENV2 NS1 (25 µg ml^−1^), followed by the addition of serial dilutions of the lysine analogue EACA (100–0 mM). Binding was detected by anti-DENV2 NS1 antibody labelling and absorbance detection at 492 nm. Data are presented as mean±sd from three independent experiments and asterisk indicates statistical significance relative to the control (no EACA) following one-way ANOVA with Dunnett’s post-hoc test (***, *P*<0.001; ****, *P*<0.0001). (**c**) Representation of the plasminogen-DENV2 NS1 complex by molecular docking. Docking solutions converged into a dominant cluster (114 models; ~57% of the water-refined structures) with a favourable score (*Z*-score=−1.3), corroborating the lysine-dependent recruitment of the viral protein (cyan) through the kringle 1 domain (KR-1; purple) in human plasminogen (grey). (**d**) Close-up view of the predicted binding between the Lys11 of one of DENV2 monomers (cyan) with the anionic and cationic centres of the lysine-binding site (purple); amino acid side chains are shown as sticks and hydrogen bonds are coloured red. (**e) and (f**) Plasminogen and uPA were mixed with glycosylated HEK293-derived DENV2 NS1 or BSA (10 µg ml^−1^), EACA (1 mM) or PBS (untreated). Plasmin activity was measured following S-2251 addition and absorbance detection at 405 nm over 1 h. (**d**) A representative graph indicating OD variation over time is shown. (**f**) OD at 60 min post-incubation is plotted according to the different conditions. Data are presented as mean±sd from three independent experiments and asterisk indicates statistical significance following one-way ANOVA with Tukey’s post-hoc test (NS, non-significant; **, *P*<0.01).

To directly confirm the interaction previously suggested by our group in the yeast two-hybrid screening [12], an immunoenzymatic assay was performed using dimeric DENV2 NS1 produced in *E. coli*. Therefore, serial dilutions of DENV2 NS1 were incubated with human plasminogen-coated microplate wells. An increase in the absorbance was observed as the DENV2 NS1 concentration increased, indicating direct binding between plasminogen and the viral protein. This was specific to plasminogen, as no DENV2 NS1-dependent increase in the OD could be detected in the BSA-coated wells (Fig. 1a). Moreover, interaction with human plasminogen was independent of the glycosylation status of the viral protein, as evidenced by employing both bacteria and mammalian cell-derived NS1 (Fig. S1, available in the online Supplementary Material).

Plasminogen is secreted by hepatocytes as a proenzyme displaying seven structural domains, four of which contain lysine-binding sites (kringle domains 1, 2, 4 and 5) responsible for target binding [24]. Hence, we sought to evaluate whether the interaction with NS1 was dependent on these same residues. Thus, in a competition assay, precoated plasminogen was incubated with fixed dimeric DENV2 NS1 concentrations and increasing concentrations of the lysine analogue EACA [35]. Increasing EACA concentrations inhibited DENV2 NS1 binding to plasminogen proportionally, evidencing that the lysine analogue was competing with the viral protein for the kringle domain(s) and suggesting that the interaction with NS1 relies on lysine-binding site(s) in plasminogen (Fig. 1b). Accordingly, molecular docking provided structural support for a lysine-dependent recruitment of DENV2 NS1 by the canonical kringle 1 anionic centre (Asp 137 and Asp 139), which represents the single lysine-binding site that remains exposed in the native plasminogen conformation (Fig. 1c, d).

Upon secretion, plasminogen adopts a closed conformation that blocks its activation loop and hinders the activity of the uPA, constituting an extra layer of control of plasminogen/plasmin function [36]. Then, fibrin/receptor binding triggers the opening of the plasminogen structure, eventually leading to its conversion into plasmin [36]. Therefore, considering that DENV2 NS1 binds human plasminogen, we hypothesized that the viral protein could also serve to activate plasminogen in a similar manner. Thus, human plasminogen was incubated with uPA in the presence or absence of DENV2 NS1, and the degree of plasminogen activation was determined by the detection of absorbance over time following chromogenic plasmin substrate (S-2251) addition. OD analysis revealed DENV2 NS1 incubation to be insufficient to trigger an enhanced plasminogen activation by uPA, as both the absorbance curve and the final plasmin activity were similar to the negative controls, wherein either no protein or BSA had been added (Fig. 1e, f). As expected, incubating plasminogen and uPA in the presence of the lysine analogue EACA enhanced plasmin formation significantly [37], and no plasmin activity could be detected in the absence of the activator, validating the experimental design (Fig. 1e, f). Therefore, our data indicate that DENV2 NS1 is unable to modulate uPA-dependent plasminogen activation despite the interaction.

### DENV2 NS1 is cleaved by human plasmin

**Fig. 2.**
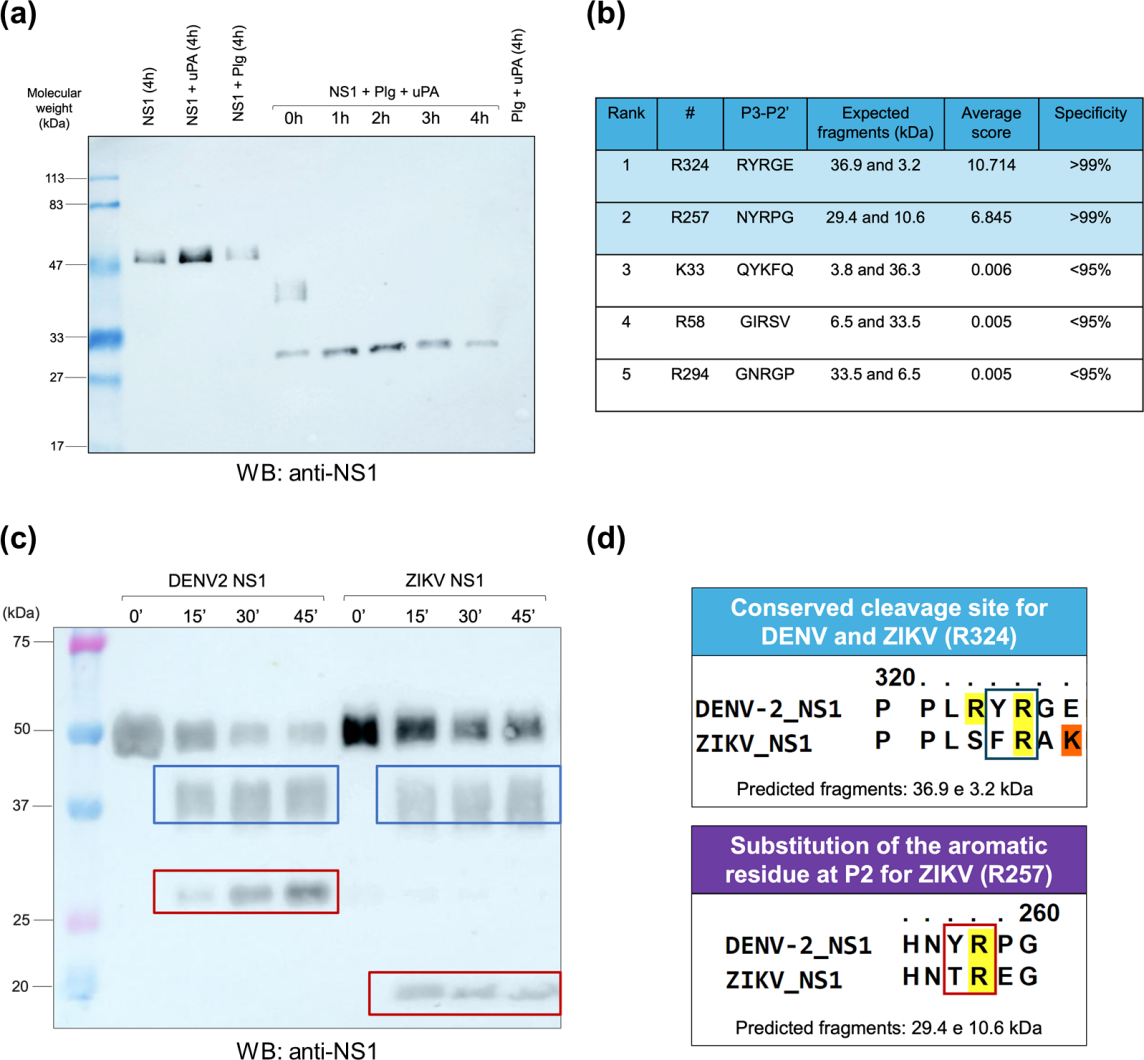
DENV2 NS1 is likely cleaved by human plasmin at arginine 257 and 324. (a) Human plasminogen (Plg; 1 µM) was incubated with uPA (200 nM) and glycosylated HEK293-derived DENV2 NS1 (1.83 µM) at 37 °C for 4 h. Samples were collected every 60 min and analysed by western blot by anti-DENV2 NS1 mouse polyclonal antibody labelling. Conditions containing only NS1, NS1 and uPA, NS1 and plasminogen or plasminogen and uPA were used as negative controls for viral protein cleavage. A representative blot out of three independent replicates is shown. (**b**) DENV2 strain 16681 NS1 amino acid sequence (UniprotKB P29991) was analysed for the prediction of human plasmin target sites through the getMerops server. Top hits were plotted and ranked according to their average score as determined by default on the server. Only the first five hits are shown. Hits with scores above the threshold (set as 1) were highlighted in light blue. Expected cleavage products for each site were predicted and are shown in kDa. (**c**) Human plasmin (1 µM) was incubated with glycosylated HEK293-derived DENV2 or ZIKV NS1 (1.83 µM) at 37 °C for 45 min, and samples were collected at 15 min intervals for subsequent western blot (WB) analysis through labelling with anti-ZIKV NS1 rabbit polyclonal antibody. Detected DENV2/ZIKV NS1 cleavage bands are highlighted in either blue or red boxes according to the target site from which they are expected to be generated, as shown in (**d**). (**d**) DENV2 and ZIKV NS1 primary sequences (UniprotKB P29991 and Q32ZE1, respectively) were aligned through PRALINE and plasmin target regions were highlighted (coloured boxes) for proper interpretation of the conservation between the two proteins (arginine and lysine residues were highlighted in yellow and orange, respectively).

Once we identified the lysine-dependent binding between NS1 and plasminogen, a common feature for plasmin substrates [36], we contemplated whether the viral protein would be a plasmin target. DENV2 NS1 was incubated with both plasminogen and uPA for 4 h. Western blot analysis revealed accumulation of lower molecular weight (MW) bands in a time-dependent manner when the viral protein was incubated with plasminogen and uPA, which was not observed in control conditions where NS1 was incubated alone or with either plasminogen or uPA (Fig. 2a), confirming NS1 cleavage over time. This effect was dependent on the active plasmin (and not only plasminogen), indicating its ability to both bind and cleave DENV2 NS1.

Following the identification of NS1 as a plasmin substrate, we attempted to identify the specific regions within the viral protein targeted by the enzyme. Previous peptide-based analyses of plasmin specificity indicated a preference for a basic residue at P1 and an aromatic side chain at the P2 position, with an additional moderate preference for lysine or hydrophobic residues at P4 [28,30]. Therefore, we screened the primary sequence of DENV2 NS1 for candidate plasmin cleavage sites, identifying two residues as a result (R324 and R257; Fig. 2b). These sites exhibited an average score of suitability to be plasmin substrates higher than the threshold (R324 and R257, score 10.174 and 6.845, respectively), as well as specificity above 99%. Interestingly, theoretical NS1 cleavage at R324 and R257 generated fragments of ~29 and ~36 kDa, respectively, similar to the MW of NS1 fragments observed in western blot assays (Fig. 2a).

To confirm whether this effect was exclusive to DENV NS1, we incubated both DENV2 and ZIKV NS1 with human plasmin. It was possible to observe that plasmin-mediated DENV2 NS1 cleavage over time generated fragments with MW like those predicted from cleavage at R257 and R324. Plasmin was also able to cleave ZIKV NS1. However, ZIKV NS1 cleavage followed a different pattern: whereas the same high MW fragment (~36 kDa) could be detected, the ~29 kDa fragment was not observed (Fig. 2c). Instead, a fragment lower than 20 kDa was observed. The R324 cleavage site, which corresponds to the configuration of basic P1 and aromatic P2 residues and generates the ~36 kDa product, seems to be conserved for both viruses. Notwithstanding, it was not the case for the R257 cleavage site, in which the Tyrosine (Y) at P2 was substituted by a Threonine (T), hinting towards a reduced affinity for the enzyme’s catalytic site (Fig. 2d). Altogether, these results show that human plasmin cleaves DENV2 NS1, likely at R257 and R324 positions, whereas for ZIKV NS1, it might occur at R324 and at another site.

### DENV2 NS1 cleavage by human plasmin attenuates viral protein-mediated pathology

**Fig. 3.**
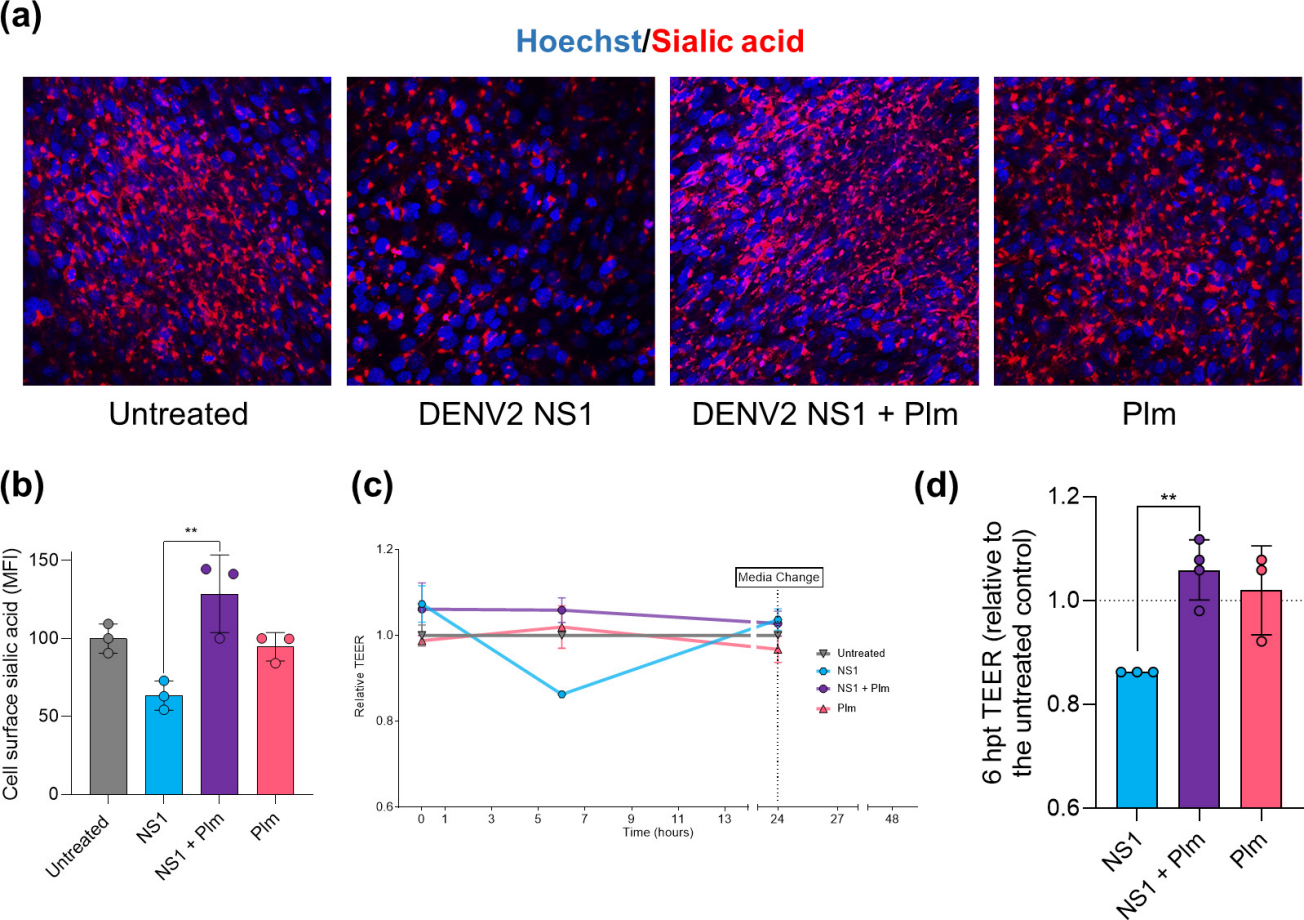
Human plasmin attenuates pathobiological effects induced by DENV2 NS1 through viral protein cleavage. (**a**) Surface sialic acid distribution (20× lens) following treatment of HPMEC cells with native or cleaved glycosylated HEK293-derived DENV2 (10 µg ml^−1^) for 6 h and staining with WGA conjugated to Alexa Fluor 647 (10 μg ml^−1^; red) and Hoechst 33342 (blue). Untreated or plasmin (Plm) treated conditions were prepared as controls. (**b**) MFI quantitation from three independent experiments in (**a**), with data presented as mean±sd and asterisks indicating significant differences based on one-way ANOVA with Tukey’s post-hoc test (**, *P*<0.01). (**c**) HPMECs were cultured on Transwell semi-permeable membranes and stimulated with either cleaved or uncleaved glycosylated HEK293-derived DENV2 NS1 (5 µg ml^−1^). The TEER was monitored over time and plotted relative to the untreated control. Media was changed 24 h post-stimulation. (**d**) Relative TEER at the lowest point of the curve for DENV2 NS1 (6 hpt) shown for all conditions. Data are presented as mean±sd from three to four independent experiments and asterisks indicate significant differences based on one-way ANOVA with Tukey’s post-hoc test (**, *P*<0.01).

NS1 is largely recognized as a key player in endothelial barrier disruption in dengue, which is partly attributed to its enhanced activation of endothelial sialidases [6,38, 39]. Thus, to assess whether plasmin-mediated cleavage might alter DENV2 NS1-mediated EGL degradation, HPMECs were stimulated with native or cleaved DENV2 NS1 and then, cell surface sialic acid, a key component of the EGL, was detected by fluorescence microscopy 6 h post-stimulation. As previously reported [6], stimulation of endothelial cell monolayers with full-length DENV2 NS1 resulted in reduced sialic acid staining, evidencing EGL disruption over time (Fig. 3a, b). Nevertheless, this effect was not observed when cells were treated with fragments of DENV2 NS1 (NS1 previously incubated with human plasmin), in which surface distribution of sialic acid was similar to that observed in the unstimulated control or plasmin treatment alone (Fig. 3a, b). Since sialic acid shedding is one of the mechanisms through which DENV2 NS1 compromises endothelial barrier integrity and human plasmin seemed to inhibit this effect, we wondered whether plasmin would attenuate NS1 induction of endothelial hyperpermeability. To verify this hypothesis, HPMECs seeded on the apical chamber of Transwells were stimulated with either native or plasmin-cleaved DENV2 NS1, and TEER was determined over time. As expected, DENV2 NS1 promoted a prominent decrease in the relative TEER, as compared to the unstimulated control, reaching a negative peak at 6 h post-stimulation (Fig. 3c, d). In contrast, HPMEC treatment with precleaved DENV2 NS1 did not induce differential barrier function of endothelial cell monolayers, which displayed similar electrical resistance over time, similar to the unstimulated control and plasmin alone (Fig. 3c, d). All together, these data evidence that plasmin-mediated cleavage of DENV2 NS1 blocks the disruptive effects of the viral protein on human endothelium.

### DENV2 NS1 enhances fibrinolysis in a mechanism that is conserved for ZIKV and WNV

**Fig. 4.**
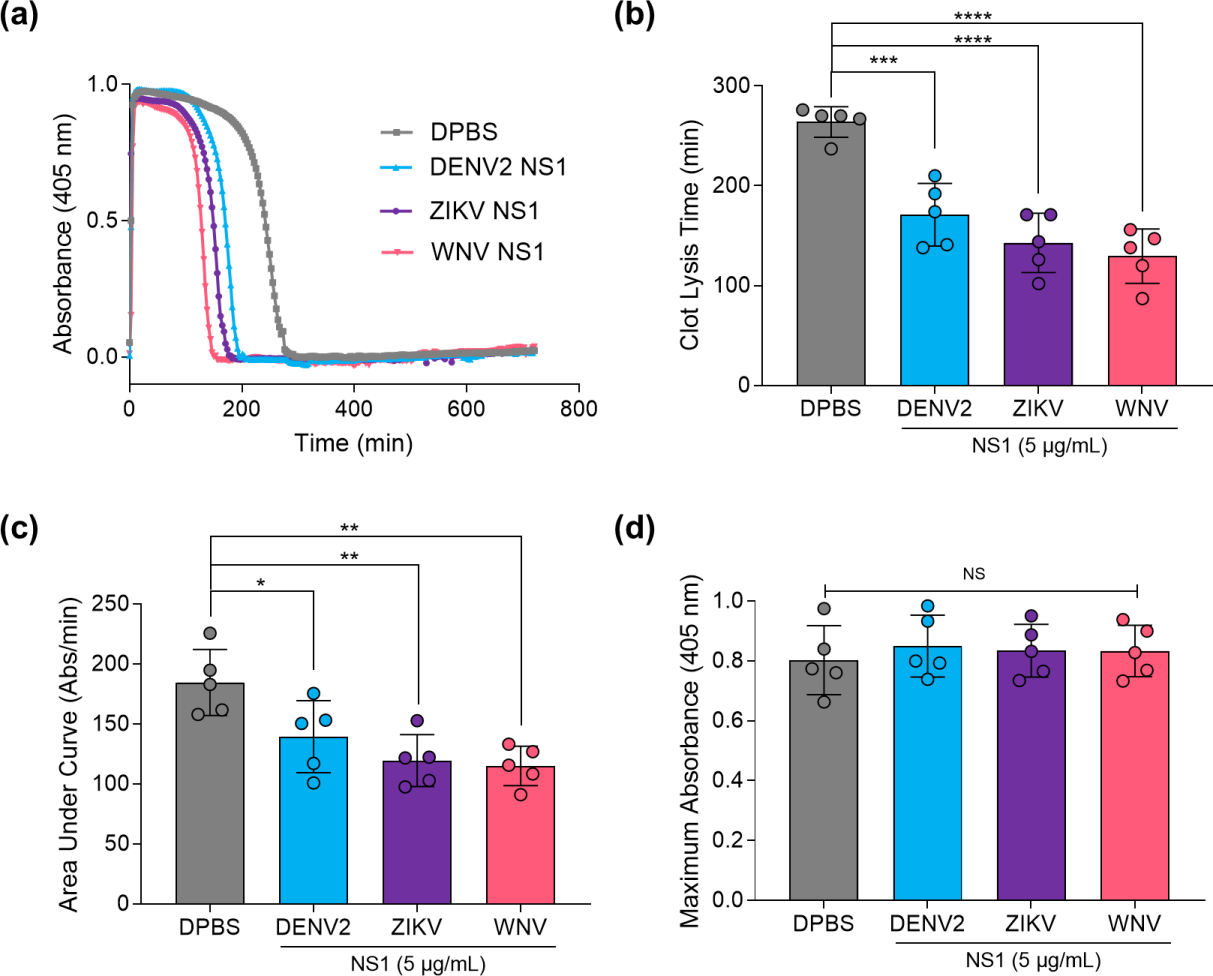
Flavivirus NS1 enhances euglobulin clot lysis catalysed by human plasmin. Euglobulin fractions obtained from plasmas of healthy donors (*n*=5 independent biological replicates) were incubated with glycosylated HEK293-derived DENV2, ZIKV or WNV NS1 (5 µg ml^−1^) on microplates. Coagulation was induced with the addition of 0.025 M CaCl_2_, and fibrinolysis, as determined by the OD kinetics, was evaluated over 720 min on the SpectraMax M5 spectrophotometer at a wavelength of 405 nm. Conditions containing only DPBS were prepared as controls. (a) Data are presented as a function of fibrin clot absorbance over time (representative curve), (b) the required time for complete clot lysis, (c) variability of absorbance per minute (Abs per min or AUC) and (d) maximum absorbance/coagulability. Data are presented as mean±sd and asterisks indicate significant differences relative to the control (DPBS; **b–d**) based on a paired one-way ANOVA with Tukey’s post-hoc test (NS, not significant; *, *P*<0.05; **, *P*<0.01; ***, *P*<0.001; ****, *P*<0.0001).

The observation that DENV2 NS1 binds human plasminogen/plasmin also suggested that it could modulate fibrinolysis, the main biological process regulated by plasmin in which uPA is not as relevant [13]. Therefore, plasma from healthy individuals was precipitated with an acetic acid solution for the harvesting of euglobulin, a fraction of peripheral blood containing low levels of fibrinolysis inhibitors. Samples were then incubated in the presence or absence of DENV2 NS1 on microplate wells, followed by addition of CaCl_2_ to trigger coagulation and monitoring of clot integrity over time as a function of absorbance. Additionally, to verify conservation of any possible effect across flaviviruses, ZIKV and WNV NS1 were also tested. All NS1 proteins promoted a faster slope in OD decrease compared to the control (Fig. 4a). Accordingly, the time for total clot lysis was significantly reduced by NS1 (Fig. 4b), which was confirmed by calculation of the area under the curve (AUC) (Fig. 4c). Notably, the presence of NS1 from different flaviviruses did not exert any influence over the maximum absorbance, indicating that the observed effect was not due to any change in coagulability but rather to the increased fibrinolytic potential (Fig. 4d). Therefore, DENV2 NS1 induces human plasmin-mediated fibrinolysis via an unknown but conserved mechanism, as this effect could be reproduced with ZIKV and WNV NS1.

## Discussion

Plasminogen is a proenzyme secreted by hepatocytes that acquires catalytic activity upon conversion into plasmin, exerting roles in fibrinolysis, wound healing and inflammation [13]. Accordingly, the plasminogen/plasmin system is tightly regulated by *α*2-antiplasmin (*α*2AP), which covalently binds to plasmin and blocks its function, as well as by the plasminogen activator inhibitors 1 and 2 (PAI-1 and PAI-2, respectively) that prevent tissue plasminogen activator (tPA) and uPA function [13]. It has been proposed that acute-phase dengue patients present an elevated tPA/PAI-1 plasma ratio [19], which may contribute to plasmin activity dysregulation during infection alongside viral envelope (E) mediated plasminogen activation [17]. Moreover, self-reactive anti-NS1 antibodies produced during infection are probably fibrinolysis inducers [40]. Anti-E protein antibodies that cross-react with plasminogen also correlate with haemorrhage and secondary infections, being associated with poorer outcomes [41]. Thus, both the direct effect of virus infection and the associated immune response mediate the bleeding disorders resulting from the abnormal activation of both fibrinolysis and coagulation in dengue patients [19,42].

Our previous identification of plasminogen as an NS1 binding partner [12] led us to further investigate the possible implications of this interaction in DENV infection. Recently, NS1 has been implicated as a key player in vascular leakage in dengue patients, directly promoting endothelial hyperpermeability and plasma extravasation [6,8]. Elucidating the molecular interplay between NS1 and plasminogen is essential for understanding haemostasis dysregulation during infection.

Our data show that DENV2 NS1 binds human plasminogen in a lysine-dependent manner, similarly to what has been shown for substrates and receptors, which also require the lysine-binding sites in the kringle domains of plasminogen/plasmin [36]. Native plasminogen usually adopts a closed conformation that renders its activation loop less accessible to its natural activators, preventing its conversion into plasmin [24]. However, fibrin/receptor binding induces plasminogen opening, allowing uPA/tPA activity [36], thus inducing plasminogen conversion into plasmin. DENV2 NS1, despite interacting with human plasminogen in a lysine-dependent manner, does not enhance plasminogen opening, exhibited by its inability to increase uPA function.

Notwithstanding, DENV2 NS1 seems to be a substrate for plasmin. Considering the profibrinolytic profile of dengue patients, with increased plasma tPA concentration [19], this result suggests that DENV NS1 is cleaved during infection, possibly affecting its bioavailability as a consequence. Plasmin is a trypsin-like serine protease that cleaves substrates at lysine/arginine residues, with substrate phage display and positional-scanning peptide libraries indicating greater cleavage efficiency when an aromatic amino acid occupies the P2 position [28,30]. Based on these occupancy restraints, our data suggest that DENV2 NS1 possesses at least two putative plasmin cleavage sites, with R324 and R257 representing the most likely target residues. Cleavage at these positions generates fragments of ~36 and 29 kDa. Interestingly, this cleavage pattern is not conserved among NS1 variants of other flaviviruses, since ZIKV NS1, upon plasmin incubation, generates the 36 kDa fragment and another fragment with MW lower than 20 kDa. It is worth noting that both R324 and R257 are located in the *β*-ladder domain (amino acids 181–352) of NS1, which faces the solvent, therefore suggesting less apparent steric hindrance for plasmin binding [18,25] and consequently its cleavage. Nonetheless, site-predicted mutagenesis approaches remain necessary for the validation of predicted cleavage sites.

DENV2 NS1 cleavage by plasmin could engender a biologically inert protein, preventing the onset of the many pathophysiological roles played by NS1 in dengue. One of these activities is its ability to induce EGL disruption [6,43]. Our data show that plasmin-cleaved DENV2 NS1 fragments are not able to promote sialic acid shedding, a hallmark of EGL disruption, in contrast to what was observed with full-length NS1. This is suggestive of a protective role of plasmin in the DENV2 NS1-induced endothelial hyperpermeability, which is partly attributed to EGL disruption [6]. This was confirmed with the observation that, following cleavage by human plasmin, the viral protein was unable to lead to a reduction in TEER, as opposed to the full-length NS1. Plasmin formation/activity in dengue [19], therefore, could limit the pathophysiological effects carried out by NS1 during infection as a result of NS1 cleavage by the protease.

Conversely, NS1 has been implicated as a promoter of bleeding in dengue [6,8,38]. Thus, the identification of DENV2 NS1 as a human plasminogen/plasmin interacting partner suggested that it could interfere with the degradation of fibrin clots, which is the main biological process regulated by intravascular plasmin [13]. It was expected that DENV2 NS1 would exert a similar effect to the lysine analogue tranexamic acid (TXA), a clinically relevant antifibrinolytic drug that inhibits fibrinolysis by competing with fibrin for plasmin substrate-binding sites [44]. However, our results show that NS1 directly induces fibrinolysis by accelerating plasmin activity, rather than inhibiting it. In the case of TXA, for every 15 min delay in its administration to bleeding patients, its effectiveness is reduced by 10% [45]. This is partly attributed to the conferred protection to plasmin against *α*2AP binding, resulting in coagulopathy by the plasmin-mediated cleavage of coagulation factors [37]. Importantly, the euglobulin fraction is poor in antifibrinolytic factors, resulting in low concentrations of *α*2AP (~7% of plasma concentration) [34], which, in turn, is correlated with the lysis time of euglobulin clots [46]. Thus, it is possible that, in this context, the concentration of NS1 used (5 µg ml^−1^), while slightly higher than that observed in the sera of dengue patients (~2 µg ml^−1^) [47], is high enough to competitively block the action of *α*2AP remnants but not to inhibit the recognition of C-terminal lysine residues in fibrin by plasmin, resulting in the induction of fibrinolysis. Therefore, it is likely that NS1 promotes the same effect *in vivo*, especially in severe dengue patients, where the fibrinolytic/coagulation control is most disbalanced [19,42]. To support this notion, it has been shown that increased tPA concentrations and elevated d-dimer (a product of fibrin degradation) correlate with bleeding in dengue patients [20], indicating that overall plasmin activity and fibrinolysis are already high during infection, being possibly further enhanced by circulating NS1. Additionally, NS1 could facilitate the bridging between fibrin and plasminogen, contributing to fibrin clot degradation through enhanced plasminogen docking onto target surfaces.

If NS1 does contribute to directing plasminogen from the circulation to fibrin clots, it is likely that the viral protein would locally favour a microenvironment where plasmin can be more readily activated, resulting in a faster clot processing. Indeed, such a profibrinolytic effect agrees well with the multifaceted but related aspects of NS1 in bleeding disorders [18], which include loss of endothelial barrier integrity (vascular leakage) [8,9], platelet activation (thrombocytopenia) [10,48] and prothrombin inhibition (dysregulation of coagulation) [11]. In this scenario, proteolytical cleavage of the viral protein might represent a mere consequence of the virus–host interactions taking place. Moreover, although fibrin-bound plasmin is resistant to inhibition, free plasmin in the circulation is efficiently inactivated by *α*2AP [36]. Therefore, the likelihood of NS1 cleavage by plasmin would be mainly determined by the control of plasminogen activation (tPA and uPA levels) as well as the consumption of *α*2AP due to enhanced plasmin generation. Thus, disease progression in dengue, which is associated with the extent of coagulation and fibrinolysis activation [19,42], might represent the balance between the profibrinolytic potential of NS1 and its detrimental cleavage by plasmin.

Finally, ZIKV and WNV NS1 seemed as strong as DENV NS1 in inducing fibrinolysis, but haemorrhage is not as common a clinical feature of these viruses as it is for DENV, which might result from multiple factors. Firstly, DENV infection has a systemic distribution, while ZIKV and WNV are associated with neuropathies [49,50], and the effect of NS1 on the endothelium reflects viral tropism [38] – that is, WNV NS1 induces hyperpermeability in brain endothelium, while DENV NS1 does so in multiple tissues. Additionally, DENV NS1 protein presents an N-terminal peptide that directs most of the viral protein expressed by infected cells to the secretory pathway, whereas in WNV, the absence of this signal results in the majority of NS1 being targeted to the plasma membrane [51]. Hence, it is likely that the inducing effect of flavivirus NS1 on fibrinolysis *in vivo* is mainly a reflection of its antigenemia and distribution during infection.

Altogether, we showed that DENV2 NS1 binds plasminogen in a lysine-dependent manner, being further targeted by active plasmin. Additionally, we show that plasmin-mediated cleavage attenuates DENV2 NS1-induced EGL degradation and endothelial hyperpermeability. Finally, NS1 accelerates tPA-dependent fibrin clot degradation, which likely results from the enhanced local plasmin formation and activity. Strikingly, this effect was also confirmed for both ZIKV and WNV NS1. This is the first time that NS1 has been shown to directly modulate fibrinolysis and suggests an additional mechanism by which NS1 contributes to haemorrhage during DENV infection.

## Supplementary material

10.1099/jgv.0.002253 Uncited Fig. S1.

## Abbreviations

AUC: area under the curve
DENV: dengue virus
DPBS: Dulbecco’s PBS
EACA: *ε*-aminocaproic acid
EGL: endothelial cell glycocalyx
ELISA: Enzyme-Linked Immunosorbent Assay
HPMEC: human pulmonary microvascular endothelial cell
hpt: h post-treatment
MFI: mean fluorescence intensity
MW: molecular weight
NS1: non-structural protein 1
PPP: poor-platelet plasma
TEER: transendothelial electrical resistance
tPA: tissue plasminogen activator
TXA: tranexamic acid
uPA: urokinase plasminogen activator
WGA: wheat germ agglutinin
WNV: West Nile virus
ZIKV: Zika virus
*α*2AP: *α*2-antiplasmin

## References

[1] Whitehead SS, Blaney JE, Durbin AP, Murphy BR (2007). Prospects for a dengue virus vaccine. *Nat Rev Microbiol*.

[2] Bhatt S, Gething PW, Brady OJ, Messina JP, Farlow AW (2013). The global distribution and burden of dengue. Nature.

[3] World Health Organization (2009). Dengue: Guidelines for Diagnosis, Treatment, Prevention and Control.

[4] Flamand M, Megret F, Mathieu M, Lepault J, Rey FA (1999). Dengue virus type 1 nonstructural glycoprotein NS1 is secreted from mammalian cells as a soluble hexamer in a glycosylation-dependent fashion. J Virol.

[5] Muller DA, Young PR (2013). The flavivirus NS1 protein: molecular and structural biology, immunology, role in pathogenesis and application as a diagnostic biomarker. Antiviral Res.

[6] Puerta-Guardo H, Glasner DR, Harris E (2016). Dengue virus NS1 disrupts the endothelial glycocalyx, leading to hyperpermeability. PLOS Pathog.

[7] Puerta-Guardo H, Biering SB, de Sousa FTG, Shu J, Glasner DR (2022). Flavivirus NS1 triggers tissue-specific disassembly of intercellular junctions leading to barrier dysfunction and vascular leak in a GSK-3β-dependent manner. Pathogens.

[8] Modhiran N, Watterson D, Muller DA, Panetta AK, Sester DP (2015). Dengue virus NS1 protein activates cells via Toll-like receptor 4 and disrupts endothelial cell monolayer integrity. Sci Transl Med.

[9] Beatty PR, Puerta-Guardo H, Killingbeck SS, Glasner DR, Hopkins K (2015). Dengue virus NS1 triggers endothelial permeability and vascular leak that is prevented by NS1 vaccination. Sci Transl Med.

[10] Quirino-Teixeira AC, Rozini SV, Barbosa-Lima G, Coelho DR, Carneiro PH (2020). Inflammatory signaling in dengue-infected platelets requires translation and secretion of nonstructural protein 1. Blood Adv.

[11] Lin S-W, Chuang Y-C, Lin Y-S, Lei H-Y, Liu H-S (2012). Dengue virus nonstructural protein NS1 binds to prothrombin/thrombin and inhibits prothrombin activation. J Infect.

[12] Silva EM, Conde JN, Allonso D, Nogueira ML, Mohana-Borges R (2013). Mapping the interactions of dengue virus NS1 protein with human liver proteins using a yeast two-hybrid system: identification of C1q as an interacting partner. PLoS One.

[13] Keragala CB, Medcalf RL (2021). Plasminogen: an enigmatic zymogen. Blood.

[14] Sulniute R, Shen Y, Guo Y-Z, Fallah M, Ahlskog N (2016). Plasminogen is a critical regulator of cutaneous wound healing. Thromb Haemost.

[15] Kamio N, Hashizume H, Nakao S, Matsushima K, Sugiya H (2008). Plasmin is involved in inflammation via protease-activated receptor-1 activation in human dental pulp. Biochem Pharmacol.

[16] Ramesh K, Walvekar VA, Wong B, Sayed AMM, Missé D (2019). Increased mosquito midgut infection by dengue virus recruitment of plasmin is blocked by an endogenous kazal-type inhibitor. *iScience*.

[17] Monroy V, Ruiz BH (2000). Participation of the dengue virus in the fibrinolytic process. Virus Genes.

[18] Glasner DR, Puerta-Guardo H, Beatty PR, Harris E (2018). The good, the bad, and the shocking: the multiple roles of dengue virus nonstructural protein 1 in protection and pathogenesis. Annu Rev Virol.

[19] Huang Y-H, Liu C-C, Wang S-T, Lei H-Y, Liu H-S (2001). Activation of coagulation and fibrinolysis during dengue virus infection. J Med Virol.

[20] Orsi FA, Angerami RN, Mazetto BM, Quaino SKP, Santiago-Bassora F (2013). Reduced thrombin formation and excessive fibrinolysis are associated with bleeding complications in patients with dengue fever: a case-control study comparing dengue fever patients with and without bleeding manifestations. BMC Infect Dis.

[21] Allonso D, da Silva Rosa M, Coelho DR, da Costa SM, Nogueira RMR (2011). Polyclonal antibodies against properly folded Dengue virus NS1 protein expressed in *E. coli* enable sensitive and early dengue diagnosis. J Virol Methods.

[22] Honorato RV, Koukos PI, Jiménez-García B, Tsaregorodtsev A, Verlato M (2021). Structural biology in the clouds: the WeNMR-EOSC ecosystem. Front Mol Biosci.

[23] Honorato RV, Trellet ME, Jiménez-García B, Schaarschmidt JJ, Giulini M (2024). The HADDOCK2.4 web server for integrative modeling of biomolecular complexes. *Nat Protoc*.

[24] Law RHP, Caradoc-Davies T, Cowieson N, Horvath AJ, Quek AJ (2012). The X-ray crystal structure of full-length human plasminogen. *Cell Reports*.

[25] Akey DL, Brown WC, Dutta S, Konwerski J, Jose J (2014). Flavivirus NS1 structures reveal surfaces for associations with membranes and the immune system. Science.

[26] Pettersen EF, Goddard TD, Huang CC, Couch GS, Greenblatt DM (2004). UCSF Chimera--a visualization system for exploratory research and analysis. J Comput Chem.

[27] Verspurten J, Gevaert K, Declercq W, Vandenabeele P (2009). SitePredicting the cleavage of proteinase substrates. Trends Biochem Sci.

[28] Hervio LS, Coombs GS, Bergstrom RC, Trivedi K, Corey DR (2000). Negative selectivity and the evolution of protease cascades: the specificity of plasmin for peptide and protein substrates. Chem Biol.

[29] Backes BJ, Harris JL, Leonetti F, Craik CS, Ellman JA (2000). Synthesis of positional-scanning libraries of fluorogenic peptide substrates to define the extended substrate specificity of plasmin and thrombin. Nat Biotechnol.

[30] Gosalia DN, Salisbury CM, Maly DJ, Ellman JA, Diamond SL (2005). Profiling serine protease substrate specificity with solution phase fluorogenic peptide microarrays. Proteomics.

[31] Zhao R, Ali G, Nie H-G, Chang Y, Bhattarai D (2020). Plasmin improves blood-gas barrier function in oedematous lungs by cleaving epithelial sodium channels. Br J Pharmacol.

[32] Simossis VA, Heringa J (2005). PRALINE: a multiple sequence alignment toolbox that integrates homology-extended and secondary structure information. Nucleic Acids Res.

[33] Schneider CA, Rasband WS, Eliceiri KW (2012). NIH Image to ImageJ: 25 years of image analysis. Nat Methods.

[34] Smith AA, Jacobson LJ, Miller BI, Hathaway WE, Manco-Johnson MJ (2003). A new euglobulin clot lysis assay for global fibrinolysis. Thromb Res.

[35] Midura-Nowaczek K, Bruzgo I, Popławski J, Roszkowska-Jakimiec W, Worowski K (1998). Effects of epsilon-aminocaproylaminoacids on fibrinolytic and caseinolytic activity of euglobulin fraction. Acta Pol Pharm.

[36] Law RHP, Abu-Ssaydeh D, Whisstock JC (2013). New insights into the structure and function of the plasminogen/plasmin system. Curr Opin Struct Biol.

[37] Longstaff C, Locke M (2019). Increased urokinase and consumption of α_2_ -antiplasmin as an explanation for the loss of benefit of tranexamic acid after treatment delay. J Thromb Haemost.

[38] Puerta-Guardo H, Glasner DR, Espinosa DA, Biering SB, Patana M (2019). Flavivirus NS1 triggers tissue-specific vascular endothelial dysfunction reflecting disease tropism. Cell Rep.

[39] Glasner DR, Ratnasiri K, Puerta-Guardo H, Espinosa DA, Beatty PR (2017). Dengue virus NS1 cytokine-independent vascular leak is dependent on endothelial glycocalyx components. PLoS Pathog.

[40] Chuang YC, Lin J, Lin YS, Wang S, Yeh TM (2016). Dengue virus nonstructural protein 1-induced antibodies cross-react with human plasminogen and enhance its activation. J Immunol.

[41] Chungue E, Poli L, Roche C, Gestas P, Glaziou P (1994). Correlation between detection of plasminogen cross-reactive antibodies and hemorrhage in dengue virus infection. J Infect Dis.

[42] Wills BA, Oragui EE, Stephens AC, Daramola OA, Dung NM (2002). Coagulation abnormalities in dengue hemorrhagic Fever: serial investigations in 167 Vietnamese children with Dengue shock syndrome. Clin Infect Dis.

[43] Wang C, Puerta-Guardo H, Biering SB, Glasner DR, Tran EB (2019). Endocytosis of flavivirus NS1 is required for NS1-mediated endothelial hyperpermeability and is abolished by a single N-glycosylation site mutation. PLoS Pathog.

[44] Kolev K, Longstaff C (2016). Bleeding related to disturbed fibrinolysis. Br J Haematol.

[45] Gayet-Ageron A, Prieto-Merino D, Ker K, Shakur H, Ageron F-X (2018). Effect of treatment delay on the effectiveness and safety of antifibrinolytics in acute severe haemorrhage: a meta-analysis of individual patient-level data from 40 138 bleeding patients. Lancet.

[46] Ilich A, Noubouossie DF, Henderson M, Ellsworth P, Betbadal KF (2020). Development and application of global assays of hyper- and hypofibrinolysis. Res Pract Thromb Haemost.

[47] Alcon S, Talarmin A, Debruyne M, Falconar A, Deubel V (2002). Enzyme-linked immunosorbent assay specific to Dengue virus type 1 nonstructural protein NS1 reveals circulation of the antigen in the blood during the acute phase of disease in patients experiencing primary or secondary infections. J Clin Microbiol.

[48] Chao C-H, Wu W-C, Lai Y-C, Tsai P-J, Perng G-C (2019). Dengue virus nonstructural protein 1 activates platelets via Toll-like receptor 4, leading to thrombocytopenia and hemorrhage. PLOS Pathog.

[49] Krauer F, Riesen M, Reveiz L, Oladapo OT, Martínez-Vega R (2017). Zika virus infection as a cause of congenital brain abnormalities and guillain-barré syndrome: systematic review. *PLoS Med*.

[50] Pierson TC, Diamond MS (2020). The continued threat of emerging flaviviruses. *Nat Microbiol*.

[51] Youn S, Cho H, Fremont DH, Diamond MS (2010). A short N-terminal peptide motif on flavivirus nonstructural protein NS1 modulates cellular targeting and immune recognition. J Virol.

