# Supplementary material for: Double-edged sword: flavivirus NS1 enhances human plasmin-mediated fibrinolysis while being targeted for cleavage and inactivation
Source: J Gen Virol. 2026 Apr 29;107(4):002253. doi: 10.1099/jgv.0.002253 (PMC13135474; doi:10.1099/jgv.0.002253)
Supplement: Uncited Fig. S1. [file jgv-107-02253-s001.pdf]

Supplementary Figure 1

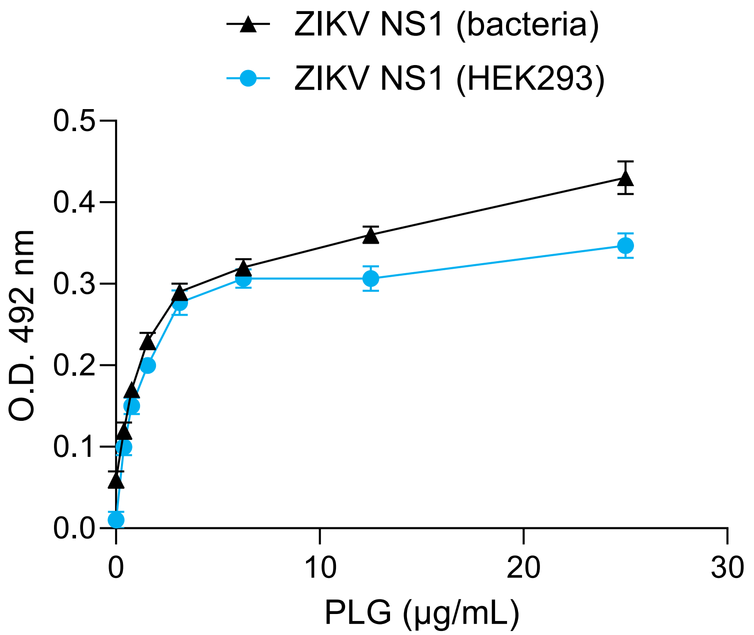

Supplementary Figure 1. Flavivirus NS1 binds human plasminogen regardless of its glycosylation status. Non-glycosylated dimeric ZIKV NS1 (bacteria) or glycosylated ZIKV NS1 (HEK293) were coated on microplate wells (10 µg/mL) and incubated with increasing concentrations of human plasminogen (0-25 µg/mL). Binding was detected by anti-plasminogen antibody labeling, followed by absorbance detection at 492 nm. Data are presented as mean ± SD from three independent experiments.
